# Emerging connectivity of programmed cell death pathways and pulmonary vascular remodelling during pulmonary hypertension

**DOI:** 10.1111/jcmm.70003

**Published:** 2024-08-17

**Authors:** Meng‐nan Yuan, Ting Liu, An‐qi Cai, Zibo Zhan, Yi‐li Cheng, Qi‐yue Wang, Yu‐xuan Xia, Nong‐er Shen, Ping Huang, Xiao‐zhou Zou

**Affiliations:** ^1^ Center for Clinical Pharmacy, Cancer Center, Department of Pharmacy, Zhejiang Provincial People's Hospital, Affiliated People's Hospital Hangzhou Medical College Hangzhou Zhejiang China; ^2^ Key Laboratory of Endocrine Gland Diseases of Zhejiang Province Zhejiang Provincial People's Hospital Hangzhou Zhejiang China; ^3^ School of Pharmaceutical Sciences Zhejiang Chinese Medical University Hangzhou Zhejiang China

**Keywords:** apoptosis, autophagy, ferroptosis, necroptosis, programmed cell death, pulmonary hypertension, pulmonary vascular remodelling, pyroptosis

## Abstract

Pulmonary hypertension (PH) is a chronic progressive vascular disease characterized by abnormal pulmonary vascular resistance and pulmonary artery pressure. The major structural alteration during PH is pulmonary vascular remodelling, which is mainly caused by the imbalance between proliferation and apoptosis of pulmonary vascular cells. Previously, it was thought that apoptosis was the only type of programmed cell death (PCD). Soon afterward, other types of PCD have been identified, including autophagy, pyroptosis, ferroptosis and necroptosis. In this review, we summarize the role of the above five forms of PCD in mediating pulmonary vascular remodelling, and discuss their guiding significance for PH treatment. The current review could provide a better understanding of the correlation between PCD and pulmonary vascular remodelling, contributing to identify new PCD‐associated drug targets for PH.

## 1 INTRODUCTION

The high mortality rate of pulmonary hypertension (PH) poses a huge challenge to clinical treatment. Programmed cell death (PCD) leads to PH‐related lesions in pulmonary vascular cells, including pulmonary arterial endothelial cells (PAECs), pulmonary arterial smooth muscle cells (PASMCs), pulmonary artery fibroblasts (PAFs) and inflammatory cells. Therefore, understanding the molecular mechanisms by which PCD regulates pulmonary vascular cells provides a theoretical basis for developing treatment strategies for PH.

Pulmonary arterial hypertension (PAH) is a class of PH characterized by elevated pulmonary vascular resistance and pulmonary artery pressure caused by pulmonary vascular remodelling. PAH can be secondary to right heart failure or even death.^1^ The prevalence of PH is about 1% in the global population and up to 10% in people over 65 years old.^2^ The prognosis for PH is poor, with 1‐, 3‐ and 5‐year survival rates of 85%, 68% and 55%. Current conventional treatments, including oxygenation, anticoagulation, diuresis, cardiac stimulation and pulmonary vasodilation, have a 1‐year mortality rate of up to 15%.^2^ The aetiology of PH involves both environmental and genetic factors, however, its pathogenesis is not fully clarified and there are no specific treatment options. Therefore, there is an urgent need to understand the pathologic changes of pulmonary vascular cells. Usually, the abnormal functional changes of vascular cells begin with apoptosis of PAECs, followed by resistance to apoptosis in PAECs, proliferation of PASMCs and activation of pulmonary artery fibroblasts (PAFs). Moreover, infiltrating inflammatory cells around pulmonary vasculature can also induce apoptosis resistance of PAECs and PASMCs, activation of PAFs, accelerating pulmonary vascular remodelling.^3^, ^4^, ^5^, ^6^

Initially, it was believed that cell death is only classified into two types: apoptosis and necrosis. Apoptosis is the earliest form of programmed cell death (PCD), which is induced by an intrinsic molecular program within cells. The apoptosis cells are accompanied by reduced cell size, membrane crinkling, chromatin condensation, formation of apoptotic vesicles and degradation of genomic DNA.^7^ Necrosis is a passive form of cell death, caused by physical or chemical damage, hypoxia and malnutrition. The necrotic cells usually show cell volume expansion, cell membrane breakage, as well as organelle expansion and fragmentation. Afterwards, other forms of PCD, including autophagy, pyroptosis, necroptosis and ferroptosis, were discovered. The main function of them is eliminating unnecessary and damaged cells under normal physiological conditions.^8^

Recently, in PH, PCD has been reported to regulate injury, inflammation, proliferation of PAECs, PASMCs, inflammatory cells and PAFs at different pathological stages of pulmonary vascular remodelling.^9^ This review summarizes the molecular biological processes of all kinds of PCD, and discusses the underlying role of PCD in regulating pulmonary vascular remodelling. The current review will provide a comprehensive understanding in different kinds of PCD in regulating pulmonary vascular remodelling, which lays an effective theoretical foundation for exploring some novel PH therapeutic strategies related to targeting PCD.

## 2 PCD PATHWAYS AND PH

### 2.1 Apoptotic pathway involved in PH

Apoptosis is a highly controlled physiological process in multicellular organisms for eliminating unwanted or abnormal cells.^10^ The pathological process of apoptosis includes membrane blebbing, cell volume condensation, rupture of nuclear membrane and breakdown of genomic DNA. The pathologic changes in apoptotic cells are regulated by many crucial genes, including proapoptotic and anti‐apoptotic proteins of the Bcl2 family, initiator caspases (e.g. caspase‐8, caspase‐9 and caspase‐10) and effector caspases (e.g. caspase‐3, caspase‐6 and caspase‐7).^11^, ^12^ Apoptosis is driven via either the intrinsic mitochondrial pathway or the extrinsic death receptor pathway, depending on the stimulus conditions. Both intrinsic and extrinsic apoptotic pathways are closely associated with PH.

#### 2.1.1 Intrinsic apoptosis and PH

##### Intrinsic apoptotic pathway

The intrinsic apoptotic pathway is primarily activated by mitochondrial outer membrane permeabilization (MOMP), which leads to the release of cytochrome c and second mitochondria‐derived activator of caspase (SMAC) into the cytoplasm.^13^ Cytochrome c binds to apoptotic protease activator factor‐1 (APF‐1) and procaspase‐9 to form apoptosomes and activate caspase‐9. Subsequently, activated caspase‐9 cleaves the precursors of caspase‐3 and caspase‐7 to release their functions, eventually leading to apoptosis.^14^ SMAC promotes apoptosis through neutralizing inhibitors of apoptosis proteins (IAPs), including X‐linked inhibitor of apoptosis protein (XIAP) and surviving.^15^ Activated caspase‐3 and caspase‐7 cleave precursors of effector apoptotic proteins to their active forms, leading to apoptosis.^16^

MOMP is always regulated by the Bcl2 protein family, which is divided into proapoptotic and anti‐apoptotic proteins.^17^ Proapoptotic proteins can be classified as MOMP effectors, activators or sensitizers. MOMP effectors, including Bcl2 antagonist/killer 1 (BAK) and Bcl2‐associated X (BAX), oligomerize on the outer mitochondrial membrane and induce pore formation, ultimately leading to MOMP.^18^ MOMP activators such as Bcl2‐like 11 (BIM), BH3‐interacting domain death agonist (BID) and p53‐upregulated binding component (PUMA) directly bind to BAK and BAX to promote their oligomerization, resulting in MOMP.^19^, ^20^ Sensitizers, including Bcl2‐associated death (BAD) and phorbol‐12‐myristate‐13‐acetate‐induced protein (NOXA), bind and inactivate specific anti‐apoptotic proteins to promote apoptosis.^21^ The transcription factor p53 is a pro‐apoptotic regulator that targets and regulates the promoter regions of several pro‐apoptotic protein genes.^22^

Anti‐apoptotic proteins, including Bcl2, B‐cell lymphoma extra‐large (Bcl‐xL) and myeloid leukaemia cell differentiation protein (Mcl‐1), possess two Bcl2 homology (BH3) domains. These two BH3 domains form a binding groove. This binding groove inhibits MOMP and intrinsic apoptosis by isolating monomer‐activated BAX and BAK or pro‐apoptotic Bcl‐2 proteins.^23^ In addition, cyclin‐dependent kinases inhibit MOMP at the transcriptional level by suppressing the expression of sensitizers and anti‐apoptotic factors.^24^ These studies suggest that proapoptotic and anti‐apoptotic proteins are balanced during apoptosis.

##### Intrinsic apoptotic pathway affecting PH

PH begins with endothelial injury, which manifests as increased apoptosis of PAECs and activation of inflammatory cells.^25^ When PH progresses to an advanced stage, PAECs, PASMCs and PAFs may develop into a state of apoptosis‐resistance and hyperproliferation.^26^ In PH, susceptibility or resistance to intrinsic apoptosis may be due to an impaired bone morphogenetic protein receptor type II (BMPRII) signalling pathway,^27^ changed levels of some growth factors and cytokines,^28^ oxidative stress,^29^ or DNA damage.^30^

###### BMPRII signalling and intrinsic apoptotic pathway

It is reported that the loss of BMPRII suppresses the expression of Bcl‐xL transcripts in PAECs, thus leading to the apoptosis of PAECs and endothelial injury. Conversely, BMPRII deletion strengthens the expression of Bcl‐xL transcripts, leading to the resistance of PASMCs to apoptosis as well as the proliferation of PASMCs.^31^ In addition, BMPRII signalling can be activated by its ligand BMP9. BMP9 can bind to the SMAD‐binding region of the BMPRII promoter, consequently reducing the early apoptosis of PAECs in PH and maintaining the stability of pulmonary vessels.^32^, ^33^ BMP9 alleviates the apoptosis of PAECs through blocking the phosphorylation of c‐Jun N‐terminal kinase (JNK), the expression of Bcl2 and the release of cytochrome c.^34^ Therefore, we speculate that the enhancement of BMPRII signalling could oppose the intrinsic apoptosis of PAECs, and alleviate early endothelial injury in PH. Targeting BMP9/SMAD/BMPRII signalling cascades may be an ideal strategy for the prevention of early PH.

###### Growth factors and cytokines and intrinsic apoptotic pathway

Increased expression of growth factors such as vascular endothelial growth factor (VEGF), platelet‐derived growth factor (PDGF) and fibroblast growth factor (FGF2) at the onset of PH can rescue endothelial cells from apoptosis.^28^ In monocrotaline‐induced PH (MCT‐PH) mice model, BIBF1000 serves as a receptor tyrosine kinase inhibitor for VEGF, PDGF and FGF, finally inhibiting the phosphorylation of AKT in the lung tissues. In addition, BIBF1000 can also inhibit the expression of proliferating cell nuclear antigen.^35^ Besides, increased protein expression of some cytokines such as TNF‐α, IL‐6, IL‐17 and IL‐1β has been observed in patients with PAH and in PH mouse models.^36^, ^37^ It has been found that miR‐125a‐5p can inhibit Bcl‐2 expression by targeting the UTR region of STAT3, thereby alleviating apoptosis resistance in PASMCs. In addition, TGF‐β1 or IL‐6 stimulation of PASMCs upregulates miR‐125a‐5p expression, whereas overexpression of miR‐125a‐5p decreases the production of TGF‐β1 and IL‐6 as well as the expression of their downstream targets STAT3 and Smad2/3.^38^, ^39^ However, the exact mechanism of how inflammatory factors regulate the apoptosis of PASMCs through microRNAs (miRNAs) is not yet elucidated.

Moreover, several inflammatory cells including macrophages, lymphocytes and neutrophils affect PH through secreting growth factors and cytokines and regulating the apoptosis of PAECs and PASMCs.^40^, ^41^, ^42^ Macrophages can be divided into classically activated macrophages (M1 macrophages), alternatively activated macrophages (M2 macrophages) and regulatory macrophages (M2b macrophages). One of the characteristics of M1 macrophages is the high expression of pro‐inflammatory factors, including IL‐1β and IL‐12, which mediate the tissue damage response.^43^ In contrast, M2 macrophages mainly express the anti‐inflammatory factor IL‐10, which can promote tissue repair.^44^ While, M2b macrophages not only secrete pro‐inflammatory factors (IL‐1β, IL‐6 and TNF‐α), but also express large amounts of the anti‐inflammatory cytokine IL‐10.^45^, ^46^ It has been found that M1 macrophages can exacerbate vascular inflammation in the initial stages of PH by accelerating apoptosis in PAECs. In contrast, M2 macrophages promoted apoptosis resistance in PAECs and PASMCs and exacerbated pulmonary vascular remodelling.^47^ Besides, M2b macrophages can also promote BAX and caspase‐9 expression in PASMCs by inhibiting the PI3K/AKT pathway, thereby alleviating the aberrant proliferation of PASMCs in MCT‐PH^46^. Donepezil is a kind of central acetylcholine inhibitor. It has been studied to promote the apoptosis of PASMCs by inhibiting M2‐macrophage activation, thereby alleviating MCT‐induced PH.^48^ T cells is also the important regulator for apoptosis in PH. It has been found that the number of CD8 T cells is significantly increased in the lung tissues of PH patients, which can exacerbate pulmonary vascular remodelling. Besides, 15‐hydroxyeicosatetraenoic acid induces PH in wild‐type mice through cytotoxic T cell‐dependent apoptosis of PAECs.^49^ Jennifer K et al. found that lymphocytes from bronchial dysplasia patients who possessed the rs278166 allele could produce a lot of endogenous NO. High levels of NO induce apoptosis and delay the development of PH.^50^ During the initiating endothelial injury phase of PH, neutrophils accumulate around the pulmonary vasculature, releasing inflammatory factors that exacerbate apoptosis and injury in PAECs.^51^ In the later stages of PH, neutrophils aggregated around the pulmonary vasculature to initiate the process of PCD and form neutrophil extracellular traps (NETs). NETs may exacerbate pulmonary vascular remodelling by promoting apoptosis resistance in PAECs and PASMCs.^52^ In addition, recent studies have found that chemokines alleviate pulmonary vascular remodelling by exacerbating or alleviating apoptosis resistance in PAECs, PASMCs, or PAFs via inflammatory cells.^6^, ^53^

###### Oxidative stress and intrinsic apoptotic pathway

Oxidative stress is another important factor affecting intrinsic apoptosis.^29^ In the advanced stages of PH, PAECs and PASMCs exhibit decreased oxidative phosphorylation and increased glycolysis.^54^, ^55^ Dichloroacetate is a metabolic regulator that increases mitochondrial oxidative phosphorylation. It has been verified that Dichloroacetate depolarized the mitochondria of PASMCs in MCT‐PH mice to release H_2_O_2_ and cytochrome c, ultimately inducing apoptosis and reducing the proliferation of PASMCs.^56^ Anoctamin1, an important chloride channel, is mainly enriched in the mitochondria. It has been reported to induce apoptosis of rat lung microvascular endothelial cells through promoting mitochondrial oxidative stress and p38 phosphorylation.^57^, ^58^ In MCT‐PH mice, mesenchymal stem cells (MSCs) therapy improved hemodynamics by alleviating pulmonary vascular remodelling.^59^ Pretreatment MSCs with PGE1 could activate the hypoxia‐inducible factor 1α pathway, reduce MSCs apoptosis, increase MSC migration to the site of lung injury.^60^ Exploration of oxidative phosphorylation‐related targets and development of oxidative phosphorylation‐related activators may be potential therapeutic approaches for PH.

###### DNA damage and intrinsic apoptotic pathway

DNA damage is also a crucial factor for intrinsic apoptosis. DNA damage mediates intrinsic apoptosis via p53. When DNA damage is severe, p53 facilitates intrinsic apoptosis through direct action on proapoptotic proteins, such as PUMA and NOXA, or through transcriptional upregulation of the proapoptotic proteins BIM, BAX and APF‐1.^61^, ^62^ In PAECs, peroxisome proliferator‐activated receptor gamma (PPARγ) and p53 mediate the transcriptional regulation of genes downstream of BMPRII.^63^ Nutlin‐3 rescues PPARγ‐p53 complex formation in BMPRII‐deficient PAECs, repairs pharmacologically induced DNA damage and ultimately prevents intrinsic apoptosis in PAECs.^64^, ^65^ It has been reported that blocking the excessive DNA damage response in PAFs reverses the apoptosis resistance of PAFs and alleviates the excessive proliferation of PAFs in idiopathic pulmonary fibrosis‐induced PH^5^.

#### 2.1.2 Extrinsic apoptosis and PH

##### Extrinsic apoptotic pathway

Extrinsic apoptosis is mainly triggered by the activation of cell death receptors, including Fas cell surface death receptor, tumour necrosis factor‐related apoptosis‐inducing ligand receptors (TRAILR1‐4) and tumour necrosis factor receptors (TNFR1/2). Upon activation by ligands, Fas and TRAILR oligomerize to form a platform, and then recruits Fas‐associated death domain protein (FADD) and procaspase‐8 to form a death‐inducing signalling complex (DISC). Activation of caspase‐8 is regulated by FLICE‐inhibitory proteins (FLIPs), which are also present in DISC. Similarly to Fas, TNFR1/2 is activated to form a platform on the cell surface to recruit TNFR1‐associated death domain protein (TRADD), receptor‐interacting protein kinase 1 (RIPK1), IAP and linear ubiquitin chain assembly complex (LUBAC), finally forming protein complexe1. Complex I, also known as the pro‐survival complex, is able to limit the pro‐apoptotic function of caspase 8 while promoting pro‐inflammatory and NFκB‐dependent signalling pathways.^66^ If IAP is absent or inhibited, RIPK1 is deubiquitinated by cylindromatosis, then forming protein complex II with FADD and caspase‐8. Similarly, complex II is regulated by FLIP, resulting in the activation of caspase‐8 and extrinsic apoptosis.

##### Extrinsic apoptotic pathway affecting PH

**FIGURE 1:**
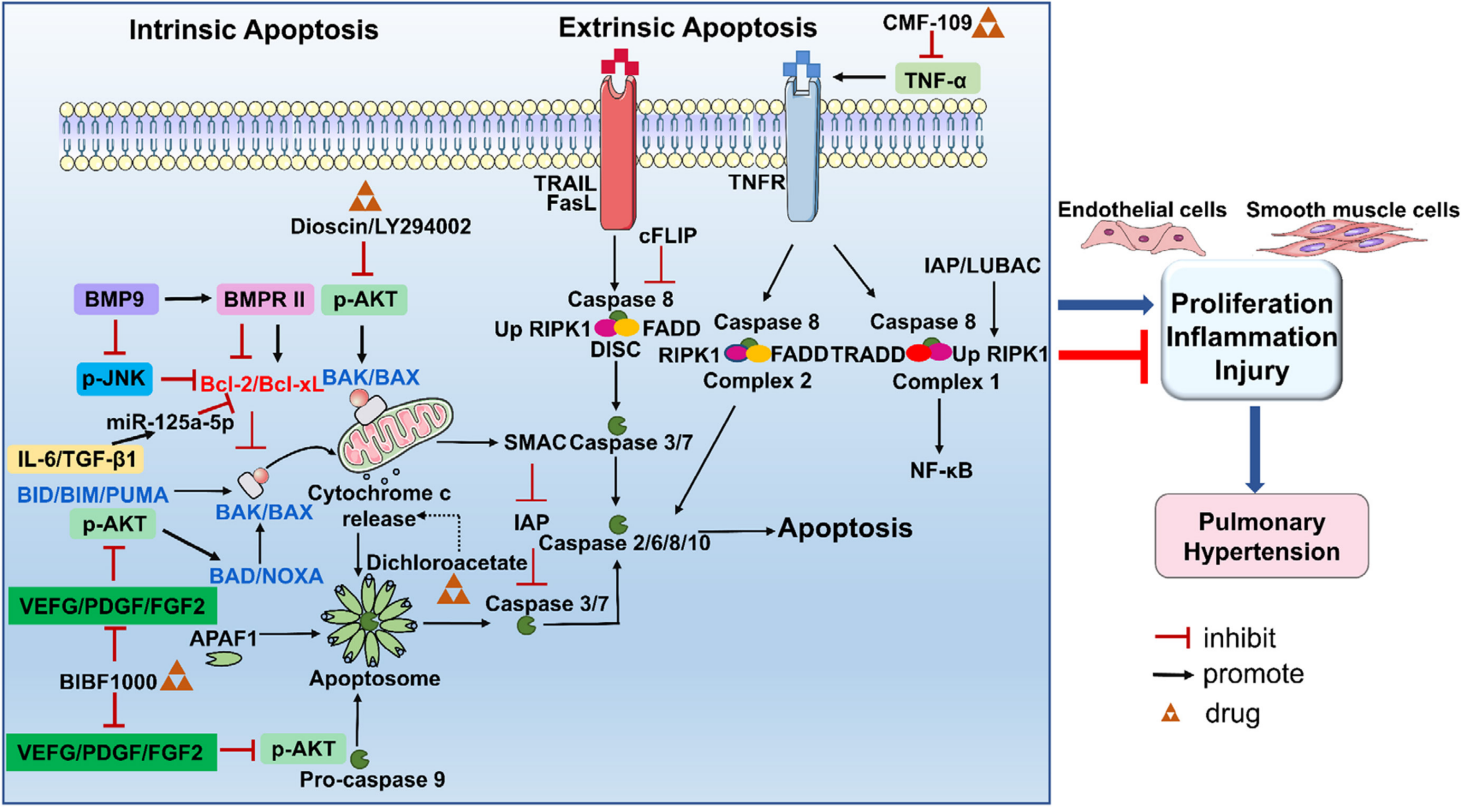
Apoptotic signalling pathways in PH.

In MCT‐PH mice, diethylcarbamazine treatment leads to a significant reduction in the expression of extrinsic apoptotic pathway markers, including FADD and caspase‐8.^67^ Apelin protein inhibitor CMF‐109, an apelin protein inhibitor, significantly prevents the apoptosis of PAECs induced by TNF‐α/CHX, further improving the early vascular dysfunction of PAH. Additionally, TNF‐α/CHX stimulates extrinsic apoptosis mainly through TNFR1, leading to the activation of JNK and MAPK pathways.^68^, ^69^ We speculated if PH can be improved via inhibition of receptors relevant to extrinsic apoptosis. Apoptotic signalling pathways in PH are shown in Figure 1.

### 2.2 Autophagic pathways involved in PH

#### 2.2.1 Autophagic pathway

Autophagy is a cellular degradation pathway that delivers cytoplasmic cargo to lysosomes. There are three main types of autophagy: macroautophagy, chaperone‐mediated autophagy and microautophagy,^70^ all of which differ in their modes of delivery to lysosomes. Macroautophagy is the most characteristic “autophagy” involving the transport of cytoplasmic cargo sequestered in double‐membrane vesicles to lysosomes.^71^ Autophagy begins with nucleation and extension of phagophores. The subsequent extension of the phagophore leads to the formation of autophagosomes with unique double‐membrane structures. Eventually, autophagosomes wrap around the cytoplasmic cargo and fuse with lysosomes, degrading the cytoplasmic cargo.^72^

Autophagy has been highly conserved during biological evolution, and its regulatory mechanisms are complex and not entirely clear. Genes associated with the regulation of cellular autophagy are known as autophagy‐related genes (ATGs). These genes encode different proteins involved in various aspects of autophagy and build a complex regulatory network. Among these, microtubule‐associated protein 1 light chain 3 (LC3), Beclin1 and p62 are key regulators, which are used as biomarkers of autophagy.^73^ During the autophagy initiation phase, the protein complex, including unc‐51‐like kinase 1 (ULK1), ATG13 and ATG101, is phosphorylated by adenosine monophosphate‐activated protein kinase (AMPK).^74^ Subsequently, phosphorylated ULK1 induces phagophore nucleation via the phosphorylation of the Beclin1/type III phosphatidylinositol 3‐kinase (PI3KIII) complex, which consists of Beclin1, ATG14, phosphoinositide‐3‐kinase regulatory subunit 4 (VPS15) and phosphatidylinositol 3‐kinase catalytic subunit type 3 (VPS34).^75^ Like Beclin1, LC3 is a homologue of ATG 8 in mammals, including LC3‐I and LC3‐II isoforms. During autophagosome formation, the LC3 precursor is hydrolyzed by ATG4 to form LC3‐I. ATG7 hydrolyzes LC3‐I to LC3‐II. LC3‐II is activated by ATG5‐ATG12‐ATG16L to couple with phosphatidylethanolamine (PE), and then binds to the phagophore membrane to promote autophagosome formation and maturation.^76^ When autophagosome formation is finished, LC3‐II is cut from PE complex by ATG4 and released back into the cytoplasm.^77^ Eventually, the autophagosome fuses with the lysosome to degrade the cytoplasmic cargo.

#### 2.2.2 Autophagic pathway affecting PH

##### Drugs, Genes and autophagic pathway

According to the existing studies, autophagy is a double‐edged sword, which may be lethal or protective for PH. HMGB1 stimulates autophagy activation by activating extracellular signal‐regulated kinase 1/2, increasing dynamin‐related protein 1 (Drp‐1) phosphorylation and Drp1‐dependent mitochondrial fission. Activated autophagy promotes the proliferation and migration of PASMCs by facilitating the degradation of BMPR II lysosomes.^78^ In addition, in MCT‐PH mice, metformin inhibited NF‐κB‐mediated autophagy through activating AMPK signalling, and then reduces the right ventricular systolic pressure and hypertrophy index.^79^ A combination of autophagy inhibitors could enhance the effect of quercetin to rescue the apoptotic resistance of PASMCs in advanced PH.^80^ *Rhodiola crenulata* extract inhibits autophagy by inhibiting the expression of LC3 and ATG7 and upregulating the expression of p62, thereby alleviating pulmonary vascular remodelling.^81^ In hypoxia‐treated PASMCs, puerarin increased the LC3‐I/LC3‐II protein ratio and decreased the expression of Beclin1 and ATG5 proteins, thereby inhibiting autophagy and proliferation of PASMCs.^82^ In PDGF‐induced PASMCs, eIF2α knockdown significantly inhibited LC3 protein expression and upregulated p62 expression, thereby inhibiting autophagy and attenuating proliferation.^83^ Paradoxically, it has been reported that piperlongumine treatment inhibits the proliferation of hypoxia‐induced PASMCs through increasing the ratio of LC3‐II/LC3‐I protein and inhibiting the expression of p62, eventually attenuating vascular remodelling and hypoxia‐induced PH^84^.

##### No coding RNA and autophagic pathway

**FIGURE 2:**
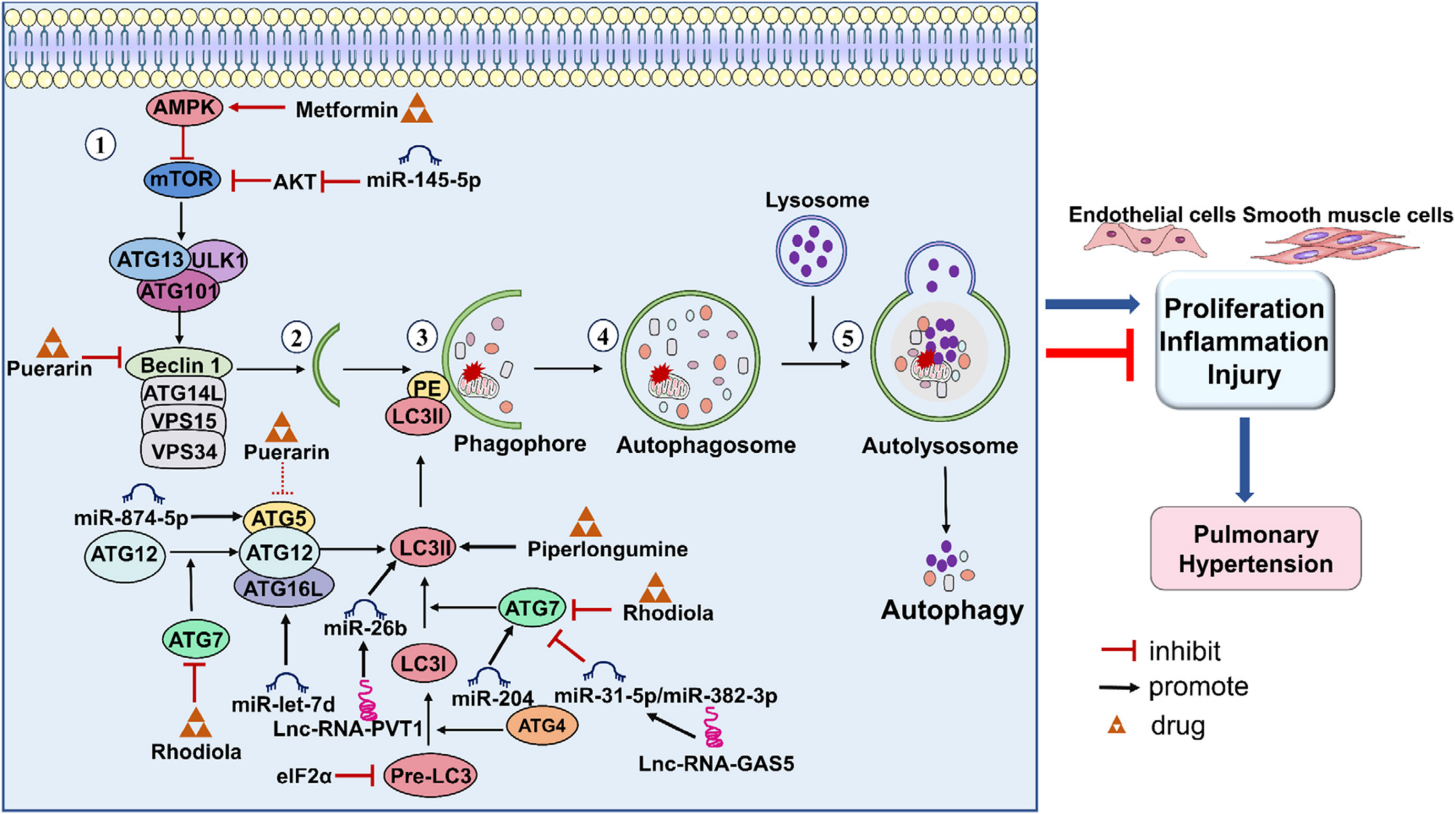
Autophagic signalling pathways in PH.

Increasing evidence suggests that miRNAs, long noncoding RNAs (lncRNAs) and circular RNAs regulate PH by targeting autophagy‐related genes. lncRNA‐GAS5 targeted miR‐382‐3p to promote autophagy in PDGF‐BB‐treated PASMCs, thereby inhibiting the viability and migration of PDGF‐BB‐treated PASMCs.^84^ Knockdown of lncRNA‐GAS5 in PAECs obviously inhibits the expression of NAT8L through targeting miR‐31‐5p, eventually moderating spermidine‐induced autophagy.^85^ In hypoxia‐induced PASMCs, inhibition of miR‐874‐5p expression in mice was followed by an increased expression of sirtuin 3 protein expression, which ultimately inhibited autophagy and hypoxia‐induced PASMCs proliferation.^86^ Similarly, lncRNA‐PVT1 expression was upregulated in PASMCs under hypoxia treatment, subsequently decreasing miR‐26b expression, and increasing the expression of CTCF, thereby enhancing autophagy.^87^ Overexpression of let‐7d in PAECs significantly inhibits autophagy of PAECs through directly inhibiting ATG16L expression, thereby reducing right ventricular systolic blood pressure and improving pulmonary hemodynamics.^88^ Downregulation of miR‐204 enhanced autophagy by targeting ATG7, thereby blocking p62‐dependent degradation of Snail and Twist, thereby inhibiting hypoxia‐induced EndMT of PAECs.^89^ Knockdown of CircSIRT1 increases the expression of miR‐145‐5p in hypoxia‐induced PASMCs, which inhibiting the expression of AKT3, repressing the autophagy of PASMCs and eventually ameliorating PH.^91^ Autophagic signalling pathways in PH are shown in Figure 2.

### 2.3 Pyroptotic pathways involved in PH

#### 2.3.1 Classical pyroptotic pathway

Pyroptosis is a form of inflammatory cell death characterized by cell swelling and membrane rupture. Rupture of the cell membrane leads to the release of cellular contents, such as interleukin‐18 (IL‐18) and interleukin‐1β (IL‐1β), eventually causing the activation of an intense inflammatory response. Pyroptosis typically begins with the activation of inflammatory sensors. To date, the widely studied inflammatory sensors include the Nod‐like receptor (NLR) family, DNA receptor absent in melanoma 2 (AIM2) and pyrin receptors. When infectious microbes release pathogen‐associated molecular patterns (PAMPs) and danger‐associated molecular patterns (DAMPs), inflammatory sensors are activated to assemble inflammasomes.^90^, ^91^

In the classical pyroptotic pathway, upon activation, inflammatory sensors oligomerize. Subsequently, oligomerized inflammatory sensors recruit the adapter apoptosis‐associated speck‐like protein, which further recruits the procaspase‐1 protein to form an inflammasome via the caspase recruitment domain (CARD).^90^ The inflammasome acts as a platform for activating caspase‐1. Activated caspase‐1 is hydrolyzed into two fragments to form a dimer, becoming mature cleaved caspase‐1. Then, the activated caspase‐1 can also cleave gasdermin D (GSDMD) to form a 31 kDa N‐terminus (N‐GSDMD) and a 22 kDa C‐terminus (C‐GSDMD). N‐GSDMD oligomerizes and perforates the plasma membrane to form pores, leading to cellular swelling, the release of inflammatory substances and ultimately pyroptosis.^92^, ^93^, ^94^ In addition, caspase‐1 can cleave the precursors of IL‐1β and IL‐18 into mature body, and then they are released from GSDMD pores to cause pyroptosis.^95^

##### Non‐classical pyroptotic pathway

In the non‐classical pyroptotic pathway, several additional caspases, including human caspases 4/5 and mouse ortholog caspase‐11, directly cleaved GSDMD in the absence of caspase‐1.^96^ Notably, caspases 4/5/11 can also be activated by direct binding to lipopolysaccharides (LPSs) through the N‐terminal CARD. Activated caspases 4/5/11 cleave GSDMD into N‐GSDMD, which oligomerizes and perforates the plasma membrane to form pores.^92^ Further, N‐GSDMD can lead to K^+^ efflux, assembling of the NLRP3 inflammasome to cause pyroptosis.^97^ Besides, caspases 4/5/11 cannot cleave the precursors of IL‐1β and IL‐18, whose maturation and secretion are dependent on NLRP3 activation due to GSDMD‐induced K^+^ efflux.^98^

Previously, apoptosis‐related caspases such as caspase‐3 and caspase‐8 were not known to stimulate gasdermin‐related proteins to induce pyroptosis. However, recently, it has been suggested that under the stimulation of TNF‐α, caspase‐8 cleaves gasdermin C (GSDMC) to produce N‐GSDMC and form pores in the cell membrane, ultimately inducing pyroptosis.^99^

#### 2.3.2 Pyroptotic pathway affecting PH

**FIGURE 3:**
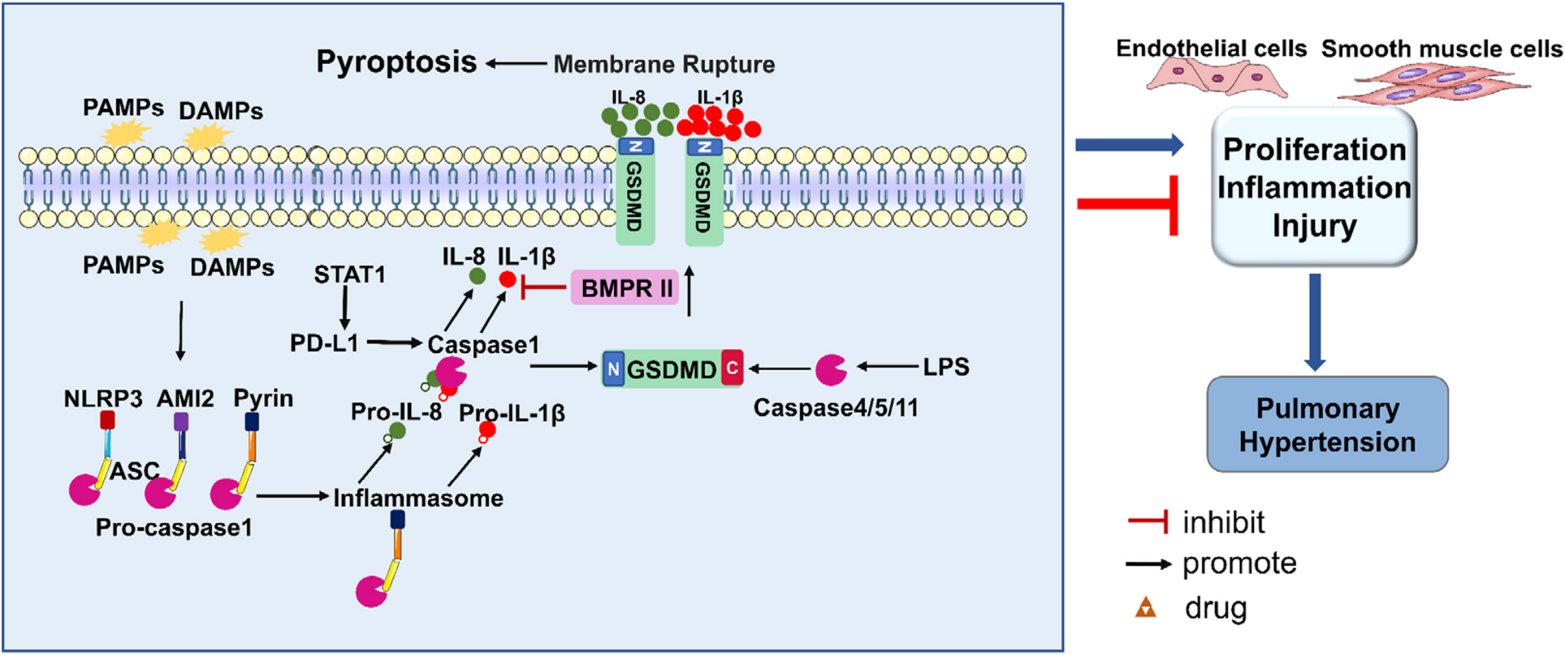
Pyroptotic signalling pathways in PH.

In MCT‐PAH mice, STAT1 promotes the upregulation of PD‐L1, which in turn activates caspase‐1 to promote pyroptosis, eventually enhancing the proliferation of PASMCs and accelerating vascular fibrosis.^100^ In systemic lupus erythematosus‐associated PH, the pulmonary arterial endothelium is severely damaged by the high level of LPS in the bloodstream. LPS was brought to endothelial cells and triggered the pyroptosis. Activated pyroptosis leads to the activation of caspase‐1, cleavage of IL‐1β, downregulation of BMPRII and expression of proinflammatory genes. Moreover, upregulation of BMPRII signalling significantly reduced the expression of IL‐8 in PAECs induced by LPS, finally preventing systemic lupus erythematosus.^101^ At present, the relationship between pyroptosis and PH is not yet fully understood. But, pyroptosis is closely associated with inflammation, which is an important inducer of PH. It is speculated that drug intervention at the different stages of pyrogenic might be a potential therapeutic strategy in PH. Pyroptotic signalling pathways in PH are shown in Figure 3.

### 2.4 Necroptotic pathways involved in PH

#### 2.4.1 Necroptotic pathway

Necroptosis is a specific regulated form of necrosis, because it is independent of caspase activity. Necroptosis begins with the activation of cellular receptors, including TNFR1, Fas cell surface death receptor and Toll‐like receptors (TLR3 and TLR4).^102^ When activated, above cellular receptors bind to the tight junction proteins in TRADD and tumour necrosis factor receptor‐associated factor 2 (TRAF2), resulting in the downstream activation of RIPK. Activated RIPK1 phosphorylates RIPK3 to form a complex that recruits and phosphorylates MLKL, eventually forming the necrosome.^103^ Necrosomes traffics with tight junction proteins to the plasma membrane, and then causes increased plasma membrane permeability, cell swelling and rupture, eventually leading to necroptosis.^104^

When necroptosis occurs, increased plasma membrane permeability leads to the formation of pores in the plasma membrane, followed by an influx of Ca^2+^ or Na^+^ ions, and the release of DAMPs, including mtDNA, HMGB1, IL‐33 and IL‐1α.^105^ Thus, necroptosis plays a significant role in the induction of inflammation. In addition, when RIPK1 is ubiquitinated, it restricts necroptosis and promotes the activation of the NF‐κB signalling pathway.^106^ Thus, necroptosis is considered a mode of inflammatory cell death, not only by the uncontrolled release of cell contents, but also by the ability to trigger NF‐κB‐dependent proinflammatory pathways.

#### 2.4.2 Necroptotic pathway affecting PH

Pulmonary vascular remodelling and inflammation in mice with MCT‐PH is accompanied by increased RIPK3 and MLKL mRNA levels. Therefore, necroptosis is considered a potential mechanism that mediates pulmonary vascular remodelling through inflammation.^107^ Recently, it was shown that in mice with MCT‐PH, RIPK3 was phosphorylated at pThr^231^/Ser^232^ in both the right ventricles and lung tissue. In the right ventricles, phosphorylation of RIPK3 may cause the increased phosphorylation of MLKL, suggesting a necroptotic cardiac injury.^108^ In addition, the elevated levels of plasma RIPK3 in rats with MCT‐PH may serve as a potential diagnostic marker for cardiac injury.^108^ Currently, the studies on necroptosis in PH only addressed the expression of the necroptosis‐associated gene RIPK3 in PH. Since RIPK3 also play a significant role in apoptosis, it is necessary to distinguish the regulation of PH by necroptosis independent of apoptosis in the following studies.

### 2.5 Ferroptotic pathways involved in PH

#### 2.5.1 Ferroptotic pathway

Ferroptosis is an iron‐dependent form of cell death, characterized by glutathione depletion, inactivation of glutathione peroxidase and the accumulation of lipid hydroperoxides. The specific morphological characteristics of ferroptosis include cell membrane rupture, enhanced mitochondrial membrane density, reduced or absent mitochondrial cristae, crumpling of the mitochondrial membrane and regular nuclear size but lack of chromatin aggregation.^109^ Ferroptosis can be activated by iron overload or inactivation of glutathione peroxidase 4 (GPX4), which directly converts lipid hydroperoxides to nontoxic lipid alcohols.^110^ Thus, iron metabolism and GPX4 activity are the two main pathways affecting susceptibility to ferroptosis.

#### 2.5.2 Ferroptotic pathway affecting PH

Recently, bioinformatics analyses of unregulated ferroptotic genes have been performed to predict targets and potential drugs. In these studies, there was a contradiction between the dysregulation and function of key ferroptosis genes such as SLA7A11.^111^, ^112^ In the PAECs from MCT‐PH mice, increased lipid peroxidation, cytosolic iron concentrations, mitochondrial damage and expression of abnormal GPX4, ferritin heavy chain 1, and NADPH oxidase 4 were followed by pulmonary artery remodelling.^113^ In addition, it is shown that the expression of solute carrier family 7 member 11 (SLC7A11) was upregulated in Sugen5416/hypoxia‐induced mice and PH patients.^114^ SLC7A11 is a subunit of the cystine/glutamate antiporter system Xc. In cancer cells, it can promote glutathione synthesis to eliminate ROS production, finally inhibiting ferroptosis. In PH, it has been reported that erastin could induce ferroptosis through inhibiting the up‐regulation of SLC7A11, ultimately reversing hypoxia‐induced proliferation of PASMCs. Although several researchers found that the inhibition of ferroptosis may be an effective treatment to alleviate PH, the underlying molecular mechanisms have not yet been explored. Elucidating the underlying mechanisms of ferroptosis regulating PH will help to develop therapeutic strategies for the treatment of PH by targeting ferroptosis.

## 3 CONCLUSIONS

In this review, we summarize recent studies to discuss the regulation of PCD during PH, as showing in Table 1. We concluded that (a) PCD, including apoptosis, autophagy, pyroptosis, necroptosis and ferroptosis, is usually associated with PH, and further studies on the specific molecular mechanisms are needed; (b) some studies have demonstrated a protective and pathological association between PCD and PH; and (c) determining PCD levels in patients with PH may be a tool for the diagnosis, treatment and prognosis of this condition.

**TABLE 1:** Effect of different forms of PCD on PH.

Forms of PCD	Target gene	Cell types	Function	Pulmonary artery remodelling (Promote or Inhibit)	Reference
Intrinsic apoptosis	Bcl2, BAK, BAX, BIM, BID, PUMA, BAD, NOXA Bcl‐xL, Mcl‐1	PAECs PASMCs PAFs macrophages lymphocytes neutrophils	Promote/inhibit injury/inflammation promote/inhibit proliferation/migration	Promote/inhibit promote/inhibit promote/inhibit	[31, 32, 33, 34, 43, 44, 45, 46, 47, 48, 49, 52, 53, 66, 67]
Extrinsic apoptosis	Caspase‐8, FADD, TRADD, TNFR1/2	PAECs	Promote injury/inflammation	Promote	[70, 71]
Autophagy	AMPK, ATG5 ATG7, ATG16L, Beclin1, LC3, LC3‐1/LC3‐II, p62	PAECs, PASMCs	Promote injury promote/inhibit proliferation/migration	Promote promote/inhibit	[80, 82, 84, 85, 86, 87, 88, 89, 91, 115, 116]
Pyroptosis	IL‐18, IL‐1β, GSDMD, Caspase‐1, Caspases 4/5/11	PAECs, PASMCs	Promote injury/inflammation promote proliferation/migration	Promote promote	[102, 103]
Necroptosis	MLKL, RIPK3	/	Promote injury/inflammation	Promote	[109, 110]
Ferroptosis	GPX4, SLC7A11	PAECs, PASMCs	Promote proliferation/migration inhibit proliferation/migration	Promote inhibit	[115, 116]

Based on these findings, we have some suggestions for future studies on PH: (a) the roles of different forms of PCD in different developmental stages of PH should be clarified; (b) the molecular mechanisms underlying different forms of PCD regulate PH should be explored; and (c) the new drugs that regulate PH by targeting different forms of PCD should be developed.

Although PCD plays a prominent role in different PH models, the pathogenesis of PH is complex and cannot be treated by the activation or inhibition of one programmed form of cell death alone. Therefore, simultaneous combination therapy of several PCD pathways may be a potential strategy for effective treatment of PH.

In conclusion, this review improves our understanding of the various functions of PCD in the pathogenesis of PH. Detailed research on the roles of PCD will help us gain a new understanding of its unknown aetiology in the future.

## AUTHOR CONTRIBUTIONS

**Meng‐nan Yuan:** Conceptualization (lead); resources (lead); writing – original draft (lead); writing – review and editing (lead). **Ting Liu:** Methodology (lead); writing – original draft (equal); writing – review and editing (equal). **An‐qi Cai:** Conceptualization (equal); investigation (equal); resources (equal). **Zibo Zhan:** Conceptualization (equal); supervision (equal). **Yi‐li Cheng:** Visualization (equal). **Qi‐yue Wang:** Investigation (equal); resources (equal). **Yu‐xuan Xia:** Investigation (equal); supervision (equal). **Nong‐er Shen:** Investigation (equal); resources (equal). **Ping Huang:** Writing – original draft (equal); writing – review and editing (lead). **Xiao‐zhou Zou:** Conceptualization (lead); writing – original draft (lead); writing – review and editing (lead).

## FUNDING INFORMATION

This study was supported by grants from the National Natural Science Foundation of China (No. 82100061, 82100059), Zhejiang Province Natural Science Foundation of China (No. LQ21H310005, LYY22H310011, LYY22H310013), Medical and Health Research Program of Zhejiang (No. 2021KY033, 2022KY078, 2022KY247), the Chinese Medicine Research Program of Zhejiang Province (No. 2022ZQ009).

## CONFLICT OF INTEREST STATEMENT

The authors declare no conflict of interest.

## Data Availability

Data sharing not applicable to this article as no datasets were generated or analysed during the current study.
